# Validity and Reliability of SCOPE (Structured Comprehensive Oral Problem-based Examination) using Generalizability and Decision Study

**DOI:** 10.12669/pjms.42.3.13939

**Published:** 2026-03

**Authors:** Mashaal Sabqat, Noorul Ain, Rehan Ahmed Khan

**Affiliations:** 1 Mashaal Sabqat, MBBS, MHPE Assistant Professor Medical Education, Islamic International Medical College, Assistant Director Riphah Institute of Assessment, Riphah International University, Islamabad, Pakistan; 2 Noorul Ain, MBBS, MHPE Assistant Professor Medical Education, Islamic International Medical College, Assistant Director Riphah Institute of Assessment, Riphah International University, Islamabad, Pakistan; 3 Rehan Ahmed Khan, MBBS, MHPE, FCPS, FRCS (Surgery), PhD ME Dean Riphah Institute of Assessment, Professor of Surgery, Department of Surgery, Riphah International University, Rawalpindi, Pakistan, Riphah International University, Islamabad, Pakistan

**Keywords:** Assessment, Generalizability study, Health professions education, Medical education, Structured viva

## Abstract

**Objective:**

To validate the SCOPE tool, determine its reliability, and identify sources of score variation using Generalizability theory, including a Decision Study to optimize its structure.

**Methodology:**

The study was conducted at Islamic International Medical College (IIMC), Rawalpindi from March 2025 to May 2025. SCOPE (Structured Comprehensive Oral Problem-based Examination) marking sheet and CVI (Content Validity Index) form were reviewed by medical educationalists for feedback and validation. Reliability was assessed through a pilot involving 37 final-year medical students. Four trained examiners conducted SCOPE, with each student assessed by one examiner using problems derived from predefined must-know and good-to-know topic lists. Performance was scored using a structured marking sheet for Recall (five marks) and Application (20 marks) categories. Reliability was assessed using Generalizability study (G-Study) with a crossover random-effects design, followed by a Decision Study (D-Study) to estimate projected reliability coefficients across varying numbers of problems and categories.

**Results:**

Ten medical educationalists provided qualitative feedback and rated the relevance and clarity of SCOPE sheet, demonstrating strong content validity (S-CVI/Ave = 0.92) and clarity (average CCA = 2.85). Reliability analysis showed a G-Coefficient value of 0.793, and Phi-coefficient value of 0.696, indicating good reliability for ranking students, and moderate reliability for absolute decisions. Students contributed the largest variance (48.13%), followed by student × problem × category interaction (19.8%). D-Study revealed that increasing the number of problems and categories substantially improved both relative and absolute reliability.

**Conclusion:**

SCOPE is a feasible, reliable and innovative tool for comparative assessments in low-resource settings, with reliability further enhanced by increasing problem numbers for each student.

## INTRODUCTION

Assessment of students is vital in determining their competence.^1^ Traditional oral assessment, or viva-voce, involves questioning candidates on a specific subject matter by one or more examiners. In medical education, it is commonly used to evaluate knowledge, decision-making, and application of theory to practice. Oral assessments also allow the assessment of a student’s confidence and reasoning under pressure.^2^ However, the format of traditional viva is typically unstructured, with examiners posing questions at their discretion. The duration of viva and the number of questions is not standardized, and the questions posed to different candidates vary.^3^ These factors can introduce bias and compromise the objectivity and fairness of the assessment. Furthermore, traditional viva is shown to suffer from poor content validity, low inter-case and inter-rater reliability, and inconsistent grading. Research indicates that approximately two-thirds of the questions in unstructured vivas test simple recall, rather than higher-order thinking.^4^,^5^

The issues of validity, reliability, and objectivity in unstructured viva exams can be addressed through structuring or standardization.^6^-^8^ In a structured viva, each examinee is presented with the same or equivalent tasks, administered under uniform conditions, within the same timeframe, with scoring being as objective as possible.^9^ Formally recognized as a separate assessment tool in medical education in 2005, structured viva has since then evolved through various documented approaches. One prominent model is the Objective Structured Viva Examination (OSVE), that utilizes a circuit of ‘stations’ to test a specific learning objective. Another approach to structuring a viva voce uses pre-designed, peer-reviewed question cards containing equitable, and progressively challenging items, with scoring guided by standardized checklists to ensure fairness.^8^,^10^,^11^ The question sets need to be updated daily when the viva spans multiple days.^3^

Developing such structured vivas requires robust question banks with detailed item categorization, requiring considerable faculty time and effort.^12^,^13^ Such demands can be particularly difficult to sustain in resource-limited settings, where faculty workload and time constraints may hinder structured viva implementtaion.^14^ To address these challenges and ensure adaptability across diverse educational contexts, we introduce the Structured Comprehensive Oral Problem-based Examination (SCOPE). SCOPE is a structured viva format designed to overcome the limitations of traditional viva, while remaining applicable across settings with varying levels of resources.

### Format of SCOPE:

The SCOPE examiners are provided with two lists of topics: ’Must-know’ and ’Should-know’. These lists are based on topic weightage, determined by multiplying impact and frequency (I x F).^15^,^16^ For each student, the examiner selects one topic from each list and develops corresponding problems/scenarios. Using these scenarios, the examiner then asks problem-based questions, advancing progressively from Recall to Application category.^16^ The problem-based nature of questions allows the assessment of students’ in-depth knowledge and reasoning, and the use of standardized topic lists ensures fairness and consistency across students.

**Fig.1:**
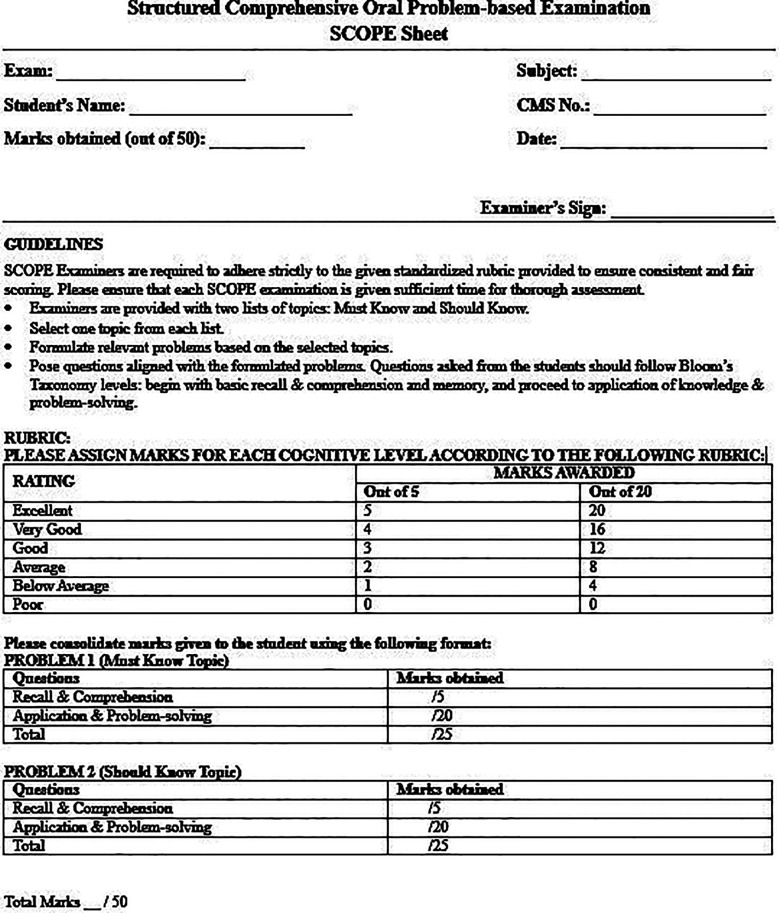
Sample SCOPE Scoring Sheet.

Scoring follows a 5-point rating scale on the SCOPE sheet (Fig.1). Each problem carries 25 marks; five for Recall category and 20 for Application category. Since each student’s SCOPE examination includes two problems, the total marks for each exam are 50. The examiners are advised to align their questioning with marks distribution to emphasize assessing application of knowledge.

### Objective of the study:

This study aims to validate the SCOPE tool and identify the sources of measurement variation in SCOPE using Generalizability theory by examining the contributions of students, problems, and categories to overall score variability. A Decision Study (D-Study) will further assess how modifying the number of problems and categories may improve reliability and optimize the SCOPE structure. The findings can support the development of an evidence-informed structured oral assessment tool suitable for diverse settings.

## METHODOLOGY

It was conducted from March 2025 to May 2025, and involved medical education experts and students from Islamic International Medical College (IIMC), Islamabad, Pakistan.

### Ethical Approval:

This study was approved by the Institutional Review Committee at Riphah International University (Appl. # Riphah/IIMC/IRC/25/1033; dated March 3, 2025).

### Phase 1 - Establishing Validity of SCOPE:

Content validity was established using the Content Validity Index (CVI). As SCOPE does not employ pre-defined items, and relies on examiner-generated questions derived from pre-specified topics, the CVI methodology was adapted. Distinct components of SCOPE, such as cognitive levels, structure of scoring rubric, and alignment of problem generation with intended constructs were defined as units of analysis and treated as “items” for validity assessment.

Eleven medical education assessment experts meeting pre-defined qualification (at least Master’s in Health Professions Education or equivalent) and experience criteria (more than five years in medical education) were invited, out of which 10 responded. The SCOPE Sheet and CVI form were emailed to these experts, who rated the item relevance and clarity on a four-point relevance *(1 = Not Relevant, 2 = Somewhat Relevant, 3 = Quite Relevant, 4 = Highly Relevant)* and a three-point clarity (1=*not clear*, 2 *=item needs some revision*; and 3=*very clear)* Likert scale, and provided qualitative feedback.

Item-level CVI (I-CVI) and scale-level CVI (S-CVI) were calculated from relevance ratings, with scores of 3-4 re-coded as 1, and 1-2 as 0. Acceptable cutoffs score were set at >0.78 for I-CVI^17^ and >0.90 for S-CVI/Ave.^18^ Content clarity average (CCA) was calculated for each item, with >80% indicating high clarity.^19^ Items below cutoff values were revised based on qualitative feedback and re-evaluated in a second round.

### Phase 2 – Determining Reliability of SCOPE:

SCOPE was piloted with 37 final-year medical students, selected via convenience sampling, at Islamic International Medical College, Rawalpindi, Pakistan, in the Department of Surgery for a formative assessment. The sample was restricted to 37 students from a cohort of 100, consistent with the pilot study design. Four examiners (Assistant Professor and above) were selected to ensure assessment expertise and underwent standardized training to minimize inter-rater variability. Each student was assessed by a single examiner.

Pilot data scores were analyzed using Generalizability Study (G-Study) to determine reliability. The object of measurement was the examinee (person, p). Two facets were examined: problems (item, i) and cognitive level being assessed (category, c: Recall/Application). Variance components and the interactions of these components were analyzed. Both problems and topics were treated as random facets to allow for generalization beyond the specific sample, and the design was crossed as each person interacted with both problems and categories.^20^ The model for this generalizability study is given below:


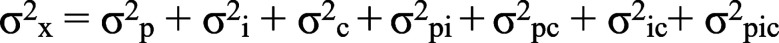


*where:* σ^2^_x_ was total variance, σ^2^_p_ was variance due to examinees, σ^2^_i_ was variance due to problems, σ^2^_c_ was variance due to categories, σ^2^_pi_ was variance due to interaction between persons and problems, σ^2^_pc_ was variance due to interaction between persons and categories, σ^2^_ic_ was variance due to interaction between problems and categories, σ^2^_pic_ was variance due to interaction between persons, problems and categories

Score percentages were used instead of raw scores to standardize data across both categories and reduce the influence of varying maximum scores on reliability analysis. SPSS version 26 was used to determine the Variance Components. The G Coefficient and Phi coefficient were computed manually for relative and absolute decisions respectively, using the following formulae:


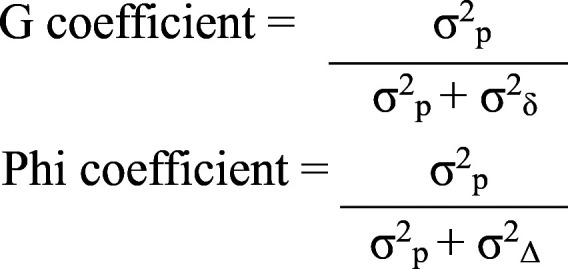


*where:* σ^2^_p_ = the variance associated with person, σ^2^_δ_ = the sum of relative error variances, σ^2^_Δ_ = the sum of absolute error variances

In G-theory, error variance is categorized into relative and absolute. Relative error variance includes sources of variability that affect the rank-ordering of students. Since all students were assessed using the same categories in SCOPE, category-related variance did not impact relative rankings and was therefore, excluded from the calculation of σ_δ_^2^. Absolute error variance includes all sources of variability affecting final scores; hence category variance was included in the calculation of σ_Δ_^2^.^20^,^21^

### Phase 3 - D Study:

A Decision Study (D-Study) was conducted to examine the effect of varying the the number of problems and categories on SCOPE reliability. Using variance components estimated from the G-Study, projected G-Coefficients (relative reliability) and Phi-Coefficients (absolute reliability) were calculated manually for different test conditions. The expected reliability coefficients were then computed to determine the optimal test design for improving SCOPE reliability.

## RESULTS

### Validation of SCOPE:

The CVI form had eight items (Table-I). In the first round, five items had an I-CVI higher than 0.79 - these were retained as such; three items (1, 4, 7) had an I-CVI equal to 0.70 - these items and their respective SCOPE components were modified according to the qualitative comments by experts. The overall content validity of the tool (S-CVI/Ave) was 0.81. After the second round, the I-CVI of items 1, 4 and 7 increased from 0.70 to 1.00, with the overall content validity of the tool (S-CVI/Ave) 0.92. The average clarity of SCOPE Sheet was 2.85, corresponding to 95% clarity (Table-I). The clarity ratings were not repeated in the second round.

**Table-I:** Relevance & Clarity ratings on SCOPE by ten experts.

Relevance Ratings																							
Items	Expert 1		Expert 2		Expert 3		Expert 4		Expert 5		Expert 6		Expert 7		Expert 8		Expert 9		Expert 10		Experts in agreement		I-CVI
***Round 1 Ratings***																							
1	1		0		0		0		1		1		1		1		1		1		7		0.7
2	1		1		1		1		0		1		1		1		1		1		9		0.9
3	1		1		1		0		1		1		1		1		1		1		9		0.9
4	1		0		1		0		0		1		1		1		1		1		7		0.7
5	1		0		0		1		1		1		1		1		1		1		8		0.8
6	1		1		1		0		1		1		1		1		1		0		8		0.8
7	1		1		1		0		1		1		1		0		1		0		7		0.7
8	1		1		1		1		1		1		1		1		1		1		10		1
																					S-CVI/Ave		0.81
***Round 2 Ratings***																							
1	1		1		1		1		1		1		1		1		1		1		10		1
2	1		1		1		1		0		1		1		1		1		1		9		0.9
3	1		1		1		0		1		1		1		1		1		1		9		0.9
4	1		0		1		1		1		1		1		1		1		1		10		1
5	1		0		0		1		1		1		1		1		1		1		8		0.8
6	1		1		1		0		1		1		1		1		1		0		8		0.8
7	1		1		1		1		1		1		1		1		1		1		10		1
8	1		1		1		1		1		1		1		1		1		1		10		1
																					S-CVI/Ave		0.92
***Clarity Ratings***																							
*Items*		*Expert 1*		*Expert 2*		*Expert 3*		*Expert 4*		*Expert 5*		*Expert 6*		*Expert 7*		*Expert 8*		*Expert 9*		*Expert 10*		*CCA*	
1		3		3		2		2		3		3		3		3		3		3		2.8	
2		3		3		3		3		3		3		3		3		3		3		3	
3		3		3		3		2		3		3		3		3		3		3		2.9	
4		3		3		3		2		3		3		3		3		3		3		2.9	
5		3		2		3		3		3		3		3		3		3		3		2.7	
6		3		3		2		2		3		3		3		3		3		3		2.8	
7		3		3		2		2		3		3		3		3		3		3		2.8	
8		3		3		2		3		3		3		3		3		3		3		2.9	
Ave CCA																						2.85	

### Reliability of SCOPE:

The overall mean score in the piloted SCOPE was 23.85±7.89 (Mean±SD). The mean scores for Problem one and Problem two were 12.16±4.47 and 11.69±4.2 respectively. Table-II lists the effects estimated in the G Study, including both the main effects (p, i, c) and the interaction effects (pi, pc, ic, pic). The G and Phi coefficients obtained by the analysis of SCOPE are given in Table-III, along with standard errors of measurement.

**Table-II:** G Study Results for SCOPE (p × i × c).

Effect	Variance Component (VC)	Percent Variance
p (Person)	180.529	48.13
i (Problem)	0.248	0.07
c (Category)	24.080	6.4
pi (Person × Problem)	56.509	15.1
pc (Person × Category)	39.366	10.5
ic (Problem x Category)	0	0
pic (Person × Problem × Category)	74.312	19.8
Total	375.044	100

**Table-III:** G and Phi coefficients.

	SCOPE
G-coefficient	0.793
SEM relative	3.55
Phi-coefficient	0.696
SEM absolute	4.30

SEM = Standard error of measurement.

### D Study Results:

The D-Study demonstrated that increasing the number of problems and categories led to improvements in both relative and absolute reliability (Table-IV). With two problems and two categories, the G-Coefficient was 0.793, and the Phi-Coefficient was 0.696. Increasing the number of problems to six raised the G-Coefficient to 0.920 and the Phi-Coefficient to 0.792. Further increasing the number of problems to 10 resulted in excellent reliability (G = 0.950, Phi = 0.815). Adding more categories also enhanced reliability but had a smaller effect compared to increasing problems.

**Table-IV:** D Study Results for SCOPE.

No. of Problems	No. of Categories	G-Coefficient	Phi-Coefficient
2	2	0.793	0.696
4	2	0.885	0.766
6	2	0.920	0.792
8	2	0.939	0.806
10	2	0.950	0.815
2	4	0.828	0.771
4	4	0.905	0.839
6	4	0.935	0.864
8	4	0.950	0.877
10	4	0.960	0.885

## DISCUSSION

This study aimed to validate the SCOPE tool, assess its reliability by determining the sources of measurement variability using Generalizability Theory (G-Theory), and conduct a Decision Study (D-Study) to ascertain how increasing the number of problems and categories could improve its reliability. The findings give insights into the strengths and limitations of SCOPE as a structured oral assessment tool and provide evidence-based recommendations for enhancing its reliability.

Our study on SCOPE reliability yielded a G-coefficient of 0.793 and a Phi-coefficient of 0.696. The G-Coefficient indicates good reliability for ranking students, with minimal error in student performance, while the Phi-coefficient reflects moderate reliability for absolute decisions (pass/fail). Relative SEM (3.55) is lower than Absolute SEM (4.30), confirming that relative rankings using SCOPE are more stable than absolute scores. This variation in reliability for absolute and relative decisions could be due to category-related variance, which impacts absolute scores by influencing performance across different categories, while relative rankings remain unaffected as all students are assessed using the same categories.

These findings suggest that while SCOPE provides moderate reliability for absolute decisions, its higher reliability for rank ordering students makes it more robust for comparative assessments. In previous studies, Cronbach’s alpha has commonly been used to assess the reliability of structured viva examinations, with values typically ranging from 0.75 to 0.88.^22^,^23^ These values are comparable to the G- and Phi-coefficient values for SCOPE found in our study. Cronbach’s alpha, while measuring internal consistency, indirectly supports both relative and absolute reliability. It ensures consistency among test items, which contributes to stable rankings and reliable pass/fail decisions. However, Cronbach’s alpha does not account for external error sources, such as problem or category variability, making it less comprehensive than G-theory.^24^

Our G-Study results show that the largest source of variance was students/examinees (48.13%). The high student variance suggests that SCOPE effectively differentiates between students based on their ability. This is consistent with the G-Study analysis of an oral language test by Shin that found examinees as the largest contributor to variability.^25^ Problem variance was minimal in our G-Study (0.07%), reflecting some variability in student performance across Recall and Application categories, also in alignment with Shin’s findings.^25^ Student × Problem interaction (15.1%) and Student × Category interaction (10.5%) indicated that students performed inconsistently across different problems and categories, as also explained by Trejo-Mejía et al in a G-Study reliability analysis of OSCE showing a notable Student × Station interaction.^26^ The Student × Problem × Category interaction (19.8%) was substantial, highlighting that some students performed differently on certain problem-category combinations. The Problem × Category interaction (0) was negligible, showing that problems were equally difficult across categories.

The D Study results found increasing the number of problems to be the most effective strategy for improving the reliability of SCOPE, particularly for absolute (for example, pass/fail) decisions. A moderate reliability could be achieved by conducting a SCOPE exam where two topics are selected from each list (Must-Know and Should-Know), totaling to four problems per student. A further increase in the number of problems, depending on the resources available, would further increase the reliability of SCOPE. This aligns with previous G-studies, which highlight that a greater number of observations per examinee reduces error variance and improves reliability.^26^-^28^

### Limitations:

This study is limited by the exclusion of examiner variability in the G-Study, as each student was assessed by a single examiner due to resource constraints and pilot nature of the design. Since examiner differences may introduce additional error, future studies should include multiple assessors per student to better evaluate variability. Although the D-Study identified the number of problems and categories for optimal reliability, the practical challenges of applying these configurations in resource-constrained settings, such as time and faculty workload, were not considered.

## CONCLUSION

SCOPE represents an innovative, feasible, and reliable assessment tool that addresses the challenges of traditional viva exams. The study’s findings validate SCOPE’s effectiveness in evaluating medical students’ knowledge and problem-solving skills. SCOPE is broadly applicable across diverse educational contexts and has the added advantage of being feasible in resource-constrained settings. It can be confidently used for comparative performance evaluations, though absolute decisions require some caution due to category-related variability. Increasing the number of problems can help improve the reliability of SCOPE.

### Recommendations:

Future studies can therefore examine the feasibility of implementing SCOPE with a greater number of problems in real-world settings. Future studies should also explore the educational impact of SCOPE to strengthen overall validation. Additional validation measures such as convergent and divergent validity should also be explored by future studies.

### Authors’ Contribution:

**MS:** Conceptualized the tool, conceived the study, collected data, analyzed it, and prepared the manuscript.

**NA:** Contributed to data analysis and prepared the manuscript.

**RAK:** Supported the conceptualization of the tool and data collection, and reviewed the manuscript.

All authors have read and approved the final version and are also responsible and accountable for the accuracy or integrity of the work.
